# Human bocavirus and human metapneumovirus in hospitalized children with lower respiratory tract illness in Changsha, China

**DOI:** 10.1111/irv.12535

**Published:** 2018-01-11

**Authors:** Jie‐ying Zhou, Ying Peng, Xiao‐you Peng, Han‐chun Gao, Ya‐ping Sun, Le‐yun Xie, Li‐li Zhong, Zhao‐jun Duan, Zhi‐ping Xie, You‐de Cao

**Affiliations:** ^1^ Department of Laboratory Medical The First People's Hospital of Hunan Chenzhou Chenzhou China; ^2^ Key Laboratory for Medical Virology Ministry of Health National Institute for Viral Disease Control and Prevention, China Center for Disease Control Beijing China; ^3^ Department of Laboratory Medical The First Affiliated Hospital of Hunan Normal University Changsha China; ^4^ Department of Paediatrics The First Affiliated Hospital of Hunan Normal University Changsha China; ^5^ Yuhang District Center for Disease Control and Prevention Hangzhou China

**Keywords:** epidemiology, human bocavirus, human metapneumovirus

## Abstract

**Background:**

Lower respiratory tract illness is a major cause of morbidity and mortality in children worldwide, however, information about the epidemiological and clinical characteristics of LRTIs caused by HMPV and HBoV in China is limited.

**Objectives:**

Human bocavirus (HBoV) and human metapneumovirus (HMPV) are two important viruses for children with lower respiratory tract infections (LRTI). We aimed to assay the correlation between viral load and clinical characteristics of HBoV and HMPV with LRTI in Changsha, China.

**Methods:**

Nasopharyngeal aspirates (NPAs) from children with LRTI were collected. Real‐time PCR was used to screen HBoV and HMPV. Analyses were performed using SPSS 16.0 software.

**Results:**

Pneumonia was the most frequent diagnosis. There was no significant difference between HBoV‐ and HMPV‐positive patients in age (*P *= .506) or hospitalization duration (*P *= .280); 24.1% and 18.2% were positive for HBoV and HMPV. HBoV infections peaked in summer (32.2%), and HMPV infections peaked in winter (28.9%). The HBoV‐positive patients had a shorter hospitalization duration than the HBoV‐negative patients (*P *= .021), and the HMPV‐positive patients had a higher prevalence of fever than the HMPV‐negative patients (*P *= .002). The HBoV viral load was significantly higher among patients aged <1 year (*P *= .006). The mean HBoV and HMPV viral loads were not significantly different between patients with single infections and coinfections. Patients infected with HBoV only were older than those coinfected with HBoV and other respiratory viruses (*P *= .005). No significant difference was found in the clinical characteristics of patients infected with HMPV only and those coinfected with HMPV and other respiratory viruses.

**Conclusion:**

Pneumonia was the most frequent diagnosis caused by HBoV and HMPV. Neither HBoV nor HMPV viral load was correlated with disease severity.

## 1 INTRODUCTION

Lower respiratory tract illness (LRTI) is a major cause of morbidity and mortality in children worldwide. Several pathogens are capable of causing LRTIs, including bacteria, viruses, and fungi. Notably, 80% of LRTIs are caused by viruses. Human metapneumovirus (HMPV) and human bocavirus (HBoV) are frequently detected in LRTIs.

HMPV, which was originally isolated in the Netherlands^1^ and is closely related to avian metapneumovirus, was the first human disease‐causing pathogen identified in the genus metapneumovirus.^2^ HMPV causes various clinical symptoms, such as cough, wheezing, and fever.^2^ Young children and the elderly are more susceptible to HMPV infection.^2^, ^3^ Indeed, HMPV mainly infects children under 5 years of age and causes upper respiratory tract and severe lower respiratory tract infections.^4^, ^5^, ^6^, ^7^, ^8^ HBoV was discovered in Sweden and classified in the family Parvoviridae,^9^ and causes various clinical symptoms, including cough, wheezing, and fever.^2^ HBoV infections are largely confined to infants and young children (<24 months). Globally, the average prevalence of HBoV in respiratory tract samples ranges from 1.0 (CI: 0.0‐2.0) to 56.8% (CI: 46.9‐66.8).^10^

However, information about the epidemiological and clinical characteristics of LRTIs caused by HMPV and HBoV in China is limited, particularly regarding the association of viral load and coinfections with clinical features and disease severity. In this study, we investigated the prevalence of HMPV and HBoV in children with LRTIs over a 2‐year period in Changsha, China. The correlation between viral load and the clinical characteristics of patients with HMPV or HBoV infections was explored.

## 2 METHODS

### 2.1 Patients and specimens

Children (<156 months of age) hospitalized for LRTI in the First Affiliated Hospital of Hunan Normal University between April 2011 and March 2013 were enrolled in the study after obtaining informed consent from their parents or guardians. All clinical and demographic data of each patient were obtained. Nasopharyngeal aspirates (NPAs) were collected within 1‐3 days of admission to hospital and placed in 2 mL of virus transport medium (Hanks’ balanced salt solution with 200 U/mL penicillin, 200 U/mL streptomycin, 200 U/mL amphotericin B, and 0.125% bovine serum albumin [BSA]). Specimens were immediately stored at −80°C and transported on dry ice to the National Institute for Viral Disease Control and Prevention, China CDC, where they were stored at −80°C until further processing. The study protocol was approved by the Ethics Committee of the First Affiliated Hospital of Hunan Normal University hospital.

### 2.2 Extraction of nucleic acid

Viral nucleic acid was extracted from 200 μL of NPA using a QIAamp MinElute Virus Spin Kit (Qiagen, Germany) according to the manufacturer's instructions. This kit enables simultaneous extraction of viral RNA and DNA.

### 2.3 Real‐time PCR for HBoV and HMPV

A One‐Step RT‐PCR Kit (Applied Biosystems, USA) was used to amplify HMPV RNA. Each 20‐μL reaction mixture comprised 10 μL of 2× RT‐PCR buffer, 0.4 μL of forward and reverse primers (20 μM), 0.4 μL of probe (10 μM), 0.8 μL of 25× RT‐PCR reverse transcriptase enzyme, 4 μL of template RNA, and 4 μL of ddH_2_O. The real‐time PCR conditions were 50°C for 30 minutes and 95°C for 10 minutes, followed by 40 cycles of 95°C for 15 seconds and 60°C for 30 seconds. TaqMan Universal Master Mix II with UNG (Applied Biosystems) was used to detect HBoV. Each 20‐μL reaction mixture comprised 10 μL of buffer, 0.4 μL of forward and reverse primers (20 μM), 0.4 μL of probe (10 μM), 0.8 μL of 25× RT‐PCR reverse transcriptase enzyme, 4 μL of template RNA, and 2.8 μL of ddH_2_O. The real‐time PCR conditions were 50°C for 2 minutes and 95°C for 10 minutes, followed by 40 cycles of 95°C for 15 seconds and 60°C for 30 seconds (Table 1). To standardize quantification of HBoV and HMPV, known numbers of DNA or RNA transcripts containing the primer targets were used in 10‐fold serial dilutions (10^0^ to 10^7^ copies/μL). Any amplification detected before 40 cycles was considered positive. Quantification of >10^0^ copies/μL (10^3^ copies/mL) was considered a positive result. An internal positive control gene (*GAPDH*), positive control, and negative (water) control were included in all reactions.

**Table 1:** Primers and probes used for the real‐time polymerase chain reaction

Assay^*a*^	Target gene^*b*^	Primer/probe sequence (5′ to 3′)^*c*^	Source or reference
HRV	5′ UTR	HRV1A,AGCCTGCGTGGCTGCCTG	38
		HRV1A2, CCTGCGTGGCGGCCARC	
		HRV1B, CCCAAAGTAGTYGGTCCCRTCC	
		HRVPb, TCCTCCGGCYCCTGAATG	
RSV	M	RSVF,GGCAAATATGGAAACATACGTGAA	39
		RSVR,TCTTTTTCTAGGACATTGTAYTGAACAG	
		RSVPb,CTGTGTATGTGGAGCCTTCGTGAAGCT	
FLUA	MP	FLUAF: GAC CRA TCC TGT CAC CTC TGA C	Internal, unpublished
		FLUAR: AGG GCA TTY TGG ACA AAK CGT CTA	
		FLUAPb: TGC AGT CCT CGC TCA CTG GGC ACG	
FLUB	Nucleoprotein	FLUBF: CCCACCRAGCAACAAAGG	Internal, unpublished
		FLUBR: CCTTCCGACATCAGCTTCACT	
		FLUBPb: CCCGGAACCCATCCCCGGA	
HMPV	F	HMPVF: CAAGTGTGACATTGCTGAYCTRAA	39
		HMPVR: ACTGCCGCACAACATTTAGRAA	
		HMPVPb: TGGCYGTYAGCTTCAGTCAATTCAACAGA	
HCOV‐NL63	Nucleoprotein	HCOV‐NL63F: GACCAAAGCACTGAATAACATTTTCC	40
		HCOV‐NL63R: ACCTAATAAGCCTCTTTCTCAACCC	
		HCOV‐NL63Pb:AACACGCT”T”CCAACGAGGTTTCTTCAACTGAG	
HCOV‐HKU1	Nucleocapsid gene	HCOV‐HKU1F: AGTTCCCATTGCTTTCGGAGTA	41
		HCOV‐HKU1R: CCGGCTGTGTCTATACCAATATCC	
		HCOV‐HKU1Pb: CCCCTTCTGAAGCAA	
HPIV1	HN	PIV1F: AGTTGTCAATGTCTTAATTCGTATCAAT	39
		PIV1R: TCGGCACCTAAGTAATTTTGAGTT	
		PIV1Pb:ATAGGCCAAAGA”T”TGTTGTCGAGACTATTCCAA	
HPIV2	HN	PIV2F: GCATTTCCAATCTACAGGACTATGA	39
		PIV2R: ACCTCCTGGTATAGCAGTGACTGAAC	
		PIV2Pb:CCATTTACC”T”AAGTGATGGAATCAATCGCAAA	
HPIV3	HN	PIV3F: TGGYTCAATCTCAACAACAAGATTTAAG	39
		PIV3R: TACCCGAGAAATATTATTTTGCC	
		PIV3Pb: CCCRTCTG”T”TGGACCAGGGATATACTACAAA	
ADV	HEXON	AdvF: GCCCCAGTGGTCTTACATGCACATC	39
		AdvR: GCCACGGTGGGGTTTCTAAACTT	
		AdvPb: TGCACCAGACCCGGGCTCAGGTACTCCGA	
HBOV	NS1	HBOV 1F: CCTATATAAGCTGCTGCACTTCCTG	42
		HBOV 1R: AAGCCATAGTAGACTCACCACAAG	
		HBOV 234F: GCACTTCCGCATYTCGTCAG	
		HBOV 3R: GTGGATTGAAAGCCATAATTTGA	
		HBOV 24R: AGCAGAAAAGGCCATAGTGTCA	
		HBOV Pb: CCAGAGATGTTCACTCGCCG	

ADV, human adenovirus; HBOV, human bocavirus 1; HPIV, human parainfluenza virus; HMPV, human metapneumovirus; HCoV, human coronavirus; HRV, human rhinovirus; FLVA, influenza virus A; FLVB, influenza virus B; RSV, respiratory syncytial virus; HN, hemagglutinin‐neuraminidase; UTR, untranslated region.

### 2.4 Detection of other viruses

Ten other respiratory viruses were screened for by real‐time PCR: human rhinovirus (HRV), adenovirus (ADV), respiratory syncytial virus (RSV), influenza viruses A and B (IFVA and IFVB), parainfluenza viruses 1‐3 (PIV1‐3), and human coronaviruses HCOV‐HKU1 and HCOV‐NL63. The real‐time PCR reagent concentrations and conditions used for the detection of all RNA viruses were identical to those for HMPV, and the concentrations and conditions for ADV were identical to those for HBoV.

### 2.5 Statistical analysis

Viral loads were expressed as initial copy numbers per real‐time PCR reaction, and quantitative results were transformed as the log number of viral copies/μL. Continuous variables are expressed as means ± standard deviation (SD) and were compared by independent samples *t* test. Categorical variables are expressed as frequencies or percentages. Comparisons were performed by chi‐squared or Fisher's exact test. Analyses were performed using SPSS 16.0 software. Two‐sided *P*‐values <.05 were considered to indicate statistical significance.

## 3 RESULTS

### 3.1 Patients’ characteristics

In total, we analyzed 1092 samples collected from LRTI patients (age 0‐13 years) during a 2‐year period. The most frequent clinical diagnoses were pneumonia (95.79%), bronchiolitis (0.91%), and bronchitis (3.30%). The male‐to‐female ratio was 1.88:1. The majority (84.2%) of the patients were aged <3 years. The age of the patients ranged from 1 day to 156 months, with a mean of 20.40 ± 24.60 months.

### 3.2 Virus identification and epidemiological characteristics

Pneumonia was diagnosed in 253 HBoV‐positive (96.2%) and 196 HMPV‐positive (98.5%) patients. The mean ages of HBoV‐ and HMPV‐positive children were 17.44 ± 21.64 and 18.76 ± 20.60 months, respectively, the difference was not significant (*P* = .506). The mean hospitalization duration of the 1092 patients was 8.08 ± 3.49 days, and those of HBoV‐ and HMPV‐positive children were 7.93 ± 3.43 and 8.28 ± 3.57 days, respectively, the difference was not significant (*P* = .280). Among patients with acute respiratory tract infection, 63.9% (168/263) of HBoV‐positive patients and 61.8% (123/199) of HMPV‐positive patients were hospitalized during their first year of life.

At least one virus was detected in 83.2% (909/1092) of the patients with LRTIs. Of the 1092 samples tested, RSV was detected in 437 (40.0%), FLVA in 78 (7.1%), FLVB in 15 (1.3%), PIV1 in 37 (3.4%), PIV2 in 61 (5.6%), PIV3 in 337 (30.9%), ADV in 228 (20.8%), HRV in 123 (11.3%), HCOV‐HKU1 in 112 (10.3%), HCOV‐NL63 in 57 (5.2%), HMPV in 199 (18.2%), and HBoV in 263 (24.1%). RSV was the virus most frequently detected in patients with respiratory infections, followed by PIV3, HBoV, ADV, and HMPV.

**Figure 1:**
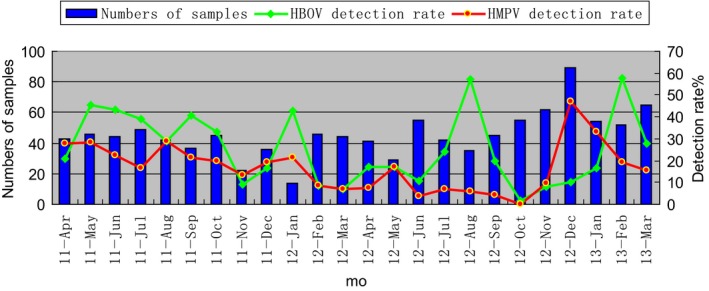
Season distribution of HBOV and HPMV detection rate during a 2‐year study

HBoV was detected in every month from April 2011 to March 2013, while HMPV was detected in every month, except for October 2012 (Figure 1). HBoV infections peaked in summer (32.2%), followed by spring (24.6%), winter (22.0%), and autumn (17.7%) (*P* = .001), while HMPV infections peaked in winter (28.9%), followed by spring (18.8%), summer (13.9%), and autumn (10.5%) (*P* = .000).

### 3.3 Clinical characteristics of HBoV‐ and HMPV‐positive patients

Patients were divided into two groups: those with and without HBoV or HMPV infections (Table 2). Cough and fever were the most frequent symptoms in HBoV‐ and HMPV‐positive patients. HBoV‐positive patients had a shorter hospitalization duration than HBoV‐negative patients (*P* = .021). There was no significant difference in the clinical features (cough, fever, tachypnea, cyanosis, O_2_ therapy, and WBC count) between HBoV‐positive and HBoV‐negative patients. HMPV‐positive patients had a higher prevalence of fever than HMPV‐negative patients (*P* = .002), while there was no significant difference in the frequency of other clinical features (cough, fever, tachypnea, cyanosis, O_2_ therapy, WBC, and hospitalization duration) between HMPV‐negative and ‐positive patients.

**Table 2:** Clinical features of HBoV‐ and HMPV‐positive patients

Clinical Data	HBoV			HMPV		
	Positive	Negative	*P*	Positive	Negative	*P*
Cough^a^	246 (93.5%)	754 (90.9%)	.205	188 (94.5%)	826 (92.5%)	.365
Fever^a^	191 (72.6%)	588 (70.9%)	.639	161 (80.9%)	653 (73.1%)	.002
Tachypnea^a^	30 (11.4%)	99 (11.9%)	.913	21 (10.6%)	108 (12.1%)	.627
Cyanosis^a^	11 (4.1%)	39 (4.7%)	.502	9 (4.5%)	41 (4.6%)	1.000
O_2_ therapy^a^	38 (15.2%)	137 (16.0%)	1.000	33 (16.6%)	142 (16.0%)	.442
WBC (×10^9^ cells/L)	10.82 ± 5.75	10.99 ± 6.10	.696	10.69 ± 6.04	10.91 ± 5.44	.610
mean ± SD^b^	n = 263	n = 829		n = 199	n = 893	
Hospitalization (mean ± SD)^b^	7.93 ± 3.43	8.71 ± 5.16	.021	8.27 ± 3.58	8.58 ± 5.04	.409

a Calculated by chi‐squared test or Fisher's exact test.

b Calculated by independent samples *t* test.

### 3.4 Viral load and clinical features

Of the patients, 24.1% (263/1092) were infected with HBoV. The log number of copies of HBoV DNA per μL ranged from 1.00 to 10.11 (median 2.72, mean 3.17 ± 1.80). Of the patients, 18.2% (199/1092) were HMPV‐positive. The log number of copies of HMPV RNA per μL ranged from 1.00 to 8.11 (median 3.21, mean 3.60 ± 1.90).

We assessed the relationship between viral load and various clinical characteristics (age, gender, respiratory rate, temperature, cyanosis, hospitalization, and WBC count) (Table 3). The HBoV viral load was significantly higher among children aged <1 year (*P* = .006); however, the other clinical characteristics were not correlated with HBoV load. HPMV load was not correlated with any of the clinical characteristics.

**Table 3:** Correlations between clinical characteristics and viral load

Clinical Data	HBoV			HMPV		
	n	Mean Viral Load	*P*	n	Mean Viral Load	*P*
Age^a^						
<1 y	168	3.40 ± 1.92	.006	99	3.46 ± 1.97	.299
≥1 y	95	2.77 ± 1.49		100	3.75 ± 1.81	
Gender^a^						
Male	184	3.25 ± 1.86	.291	125	3.74 ± 1.95	.174
Female	79	2.99 ± 1.64		74	3.36 ± 1.78	
Respiratory rate^a^						
Normal	30	3.66 ± 2.24	.122	21	3.44 ± 1.56	.521
High	233	3.11 ± 1.73		178	3.65 ± 1.98	
Temperature^a^						
Normal	72	2.85 ± 1.28	.077	36	3.21 ± 1.63	.161
Fever	191	3.29 ± 1.94		163	3.70 ± 1.94	
Cyanosis^a^						
No	252	3.51 ± 2.35	.519	190	3.64 ± 1.91	.148
Yes	11	3.15 ± 1.77		9	2.71 ± 1.31	
Hospitalization^a^						
<7 d	93	3.00 ± 1.54	.269	27	3.62 ± 2.01	.916
≥7 d	170	3.26 ± 1.92		78	3.59 ± 1.85	
WBC^a^						
≤10 (×10^9^ cells/L)	143	3.27 ± 1.80	.325	107	3.77 ± 2.11	.180
>10 (×10^9^ cells/L)	120	3.05 ± 1.80		92	3.41 ± 1.60	

a Calculated by independent samples *t* test.

### 3.5 Coinfections with other viruses

**Figure 2:**
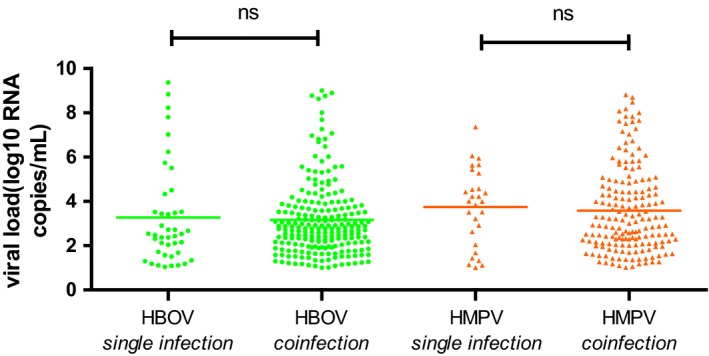
Viral loads of NPAs from patients with or without respiratory coinfections

Of the HBoV‐ and HMPV‐positive patients, 83.7% (220/263) and 85.9% (171/199), respectively, were coinfected with other respiratory viruses. The viruses most frequently detected in coinfections with HBoV were RSV (n = 124), PIV3 (n = 113), ADV (n = 69), and HMPV (n = 52). The viruses most frequently detected in coinfections with HMPV were RSV (n = 86), PIV3 (n = 85), HBoV (n = 52), and ADV (n = 49). HBoV and HMPV mean values of viral load were not significantly different between single infections and coinfections (*P* = .764 and *P* = .082) (Figure 2). Patients infected with HBoV only were older than those coinfected with HBoV and other respiratory viruses (*P* = .005) (Table 4). There were no significant differences in the clinical characteristics of patients infected with HMPV and those coinfected with HMPV and other respiratory viruses (Table 5).

**Table 4:** Clinical characteristics of patients infected with HBoV only and those coinfected with HBoV and other respiratory viruses

Clinical Data	Single HBoV Infection (n = 43)	HBoV Coinfection (n = 220)	*P*
Males^a^	31 (72.1%)	153 (69.6%)	.856
Months ± SD^b^	26.05 ± 31.22	15.89 ± 18.94	.005
Fever^a^	29 (67.4%)	162 (73.6%)	.717
Tachypnea^a^	4 (9.3%)	26 (11.8%)	.796
Cyanosis^a^	2 (4.7%)	9 (4.1%)	1.000
O_2_ therapy^a^	6 (14.0%)	32 (14.5%)	.806
Hospitalization^b^	7.67 ± 2.82	7.96 ± 3.54	.615
WBC^b^	11.64 ± 6.91	10.64 ± 5.5	.301
Diagnosis^b^			
Bronchitis	3 (7.0%)	7 (3.2%)	.213
Pneumonia	40 (93.0%)	213 (96.8%)	.213

a Calculated by chi‐squared test or Fisher's exact test.

b Calculated by independent samples *t* test.

**Table 5:** Clinical characteristics of patients infected with HMPV only and those coinfected with HMPV and other respiratory viruses

Clinical data	Single HMPV (n = 40)	HMPV coinfections (n = 159)	*P*
Males^a^	28 (70.0%)	97 (61.00%)	.361
Months ± S D^b^	18.86 ± 24.06	19.00 ± 19.91	.197
Fever^a^	33 (82.50%)	128 (80.50%)	1.000
Tachypnea^a^	5 (12.5%)	16 (10.1%)	.773
Cyanosis^a^	3 (7.5%)	6 (3.2%)	.388
O_2_ therapy^a^	5 (12.5%)	28 (17.6%)	.634
Hospitalization^b^	8.15 ± 2.43	8.32 ± 3.81	.786
WBC^b^	10.94 ± 5.42	10.65 ± 6.18	.787
Diagnosis^b^			
Bronchitis	1 (2.5%)	2 (5.0%)	.492
Pneumonia	39 (97.5%)	157 (98.7%)	.492

a Calculated by chi‐squared test or Fisher's exact test.

b Calculated by independent samples *t* test.

### 3.6 Viral load and disease severity

**Figure 3:**
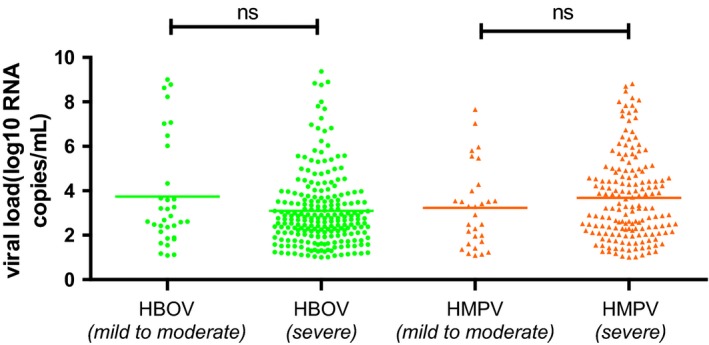
Viral loads of NPAs from patients with mild‐to‐moderate and severe pneumonia

According to the British Thoracic Society Guidelines for the Management of Community‐Acquired Pneumonia in Children^11^ and the clinical diagnosis, LRTIs were classified as mild to moderate or severe. The mean HBoV and HMPV viral loads did not differ significantly between the two groups (*P* = .053 and *P* = .231) (Figure 3).

## 4 DISCUSSION

In this study, we used real‐time PCR to explore the etiology and examine the correlations between viral load and the clinical features of pediatric HBoV and HMPV LRTIs in Changsha, China, over two consecutive years. Pneumonia was the most frequent diagnosis in HBoV‐positive patients (96.2%) and HMPV‐positive patients (98.5%) with LRTIs, which is in agreement with previous reports,^12^, ^13^, ^14^, ^15^ Calvo reported that HMPV‐positive patients were younger than HBoV‐positive patients; in contrast, the ages of HBoV‐ and HMPV‐positive patients did not differ significantly in our study. HMPV and HBoV frequently resulted in hospitalization,^8^, ^16^ which has been reflected in our results. There was no difference in the mean hospitalization duration of HBoV‐positive (7.93 ± 3.43 days) and HMPV‐positive (8.28 ± 3.57 days) patients. This is not in agreement with the study by Calvo, in which hospitalization duration was longer in patients with HMPV. However, our data are in line with the findings of Davis.^8^ Our findings suggest that more attention should be paid to young children with LRTIs due to the considerable hospitalization duration. In our study, HBoV and HMPV infections were almost completely confined to infants and children; 63.9% (168/263) of HBoV patients and 61.8% (123/199) of HMPV patients were hospitalized during their first year of life, in agreement with the study by Edwards.^17^ This is likely due to the weak immunity of children aged <1 year.

At least one virus was detected in 83.2% (909/1092) of patients with LRTIs. This is higher than previous study (62.34%) in Changsha that used traditional PCR,^18^ and lower than that a study in Lanzhou that used real‐time PCR.^6^ On the one hand, the high detection rate in our study could be caused by the greater sensitivity of real‐time PCR compared with traditional PCR. On the other hand, it is possible that the detection rate differs geographically. RSV was the most frequently virus detected in patients with respiratory infections, followed by PIV3, HBoV, ADV, and HMPV. Our data support previous reports of RSV, PIV3, HBoV, ADV, and HMPV as the major agents associated with LRTIs among children in a hospital setting.^12^, ^19^, ^20^, ^21^, ^22^ The HBoV infection rate (24.1%) in the present study is consistent with earlier reports (1.9%‐24.6%^8^, ^9^, ^16^, ^23^, ^24^, ^25^, ^26^), and the incidence of HMPV (18.2%) in patients with respiratory tract infections is similar to that in other regions (1.5%‐18%^2^, ^9^, ^17^, ^23^, ^26^, ^27^, ^28^). Therefore, HMPV and HBoV are major causes of LRTIs worldwide.

The seasonal peaks of HBoV and HMPV infections vary among countries because of differences in climatic and geographic factors. In this study, HBoV activity peaked in summer, in agreement with the report by Jiang.^29^ In contrast, detection of HBoV in Lanzhou peaked in December and April,^30^ possibly due to the dry, cold weather in Lanzhou and the warm, humid weather in Changsha. HMPV detection peaked in winter, which is in line with previous studies.^2^, ^7^, ^18^, ^31^ In contrast, in Hong Kong, HMPV detection peaks in spring/summer.^32^ The seasonality of HBoV differed geographically, possibly due to climatic factors.

The most frequent symptoms of HBoV‐ and HMPV‐positive patients were cough and fever, in accordance with previous reports.^6^, ^18^, ^33^ However, Deng reported that in Chongqing, wheezing was the most frequent symptom exhibited by HBoV‐positive patients with severe LRTIs.^16^ There was no difference between the HBoV‐ and HMPV‐positive patients in the incidence of fever, tachypnea, cyanosis, O_2_ therapy, or WBC count. The HBoV‐positive patients had a shorter hospitalization duration than HBoV‐negative patients (*P* = .021). In contrast, Deng reported that HBoV‐positive patients had a longer hospitalization duration. The longer hospitalization duration of HBoV‐negative patients in our study may have been caused by the presence of other viruses (such as RSV and PIV3) in many of them. Also, hospitalization duration was significantly associated with age (≤6 months), maternal smoking during pregnancy, and a family history of asthma.^19^ A high HMPV viral load contributes to development of fever.^4^, ^5^ Indeed, HMPV‐positive patients had a higher incidence of fever than HMPV‐negative patients (*P* = .002) in our study.

We assessed the relationship between viral load and clinical features (age, gender, respiratory rate, temperature, cyanosis, hospitalization duration, and WBC count). The only significant association of HBoV‐ or HPMV‐positive patients was a higher viral load in <1‐year‐old HBoV‐positive patients. This is in agreement with Jiang's report that patients with a high viral load were significantly younger.^29^ In contrast, the duration of wheezing and hospitalization was longer in children with a high than a low HBoV viral load in the study by Deng,^16^ possibly due to inclusion of only patients with severe LRTIs.

HMPV and HBoV coinfections with other viruses are common in children. In this study, 83.7% (220/263) of HBoV‐positive patients and 85.9% (171/199) of HMPV‐positive patients had mixed infections. Both the HBoV (83.7%) and HMPV (85.9%) coinfection rate was higher than reported previously (17%‐75%^13^, ^25^, ^34^ and 81.8%,^34^ respectively). Similarly, HMPV and HBoV are frequently present as mixed infections.^19^, ^20^, ^22^ RSV was the virus detected most frequently in coinfections with HBoV and HBoV, as reported previously.^6^, ^16^ Viral load did not differ significantly between single and mixed HBoV and HMPV infections, in agreement with the study by Neske.^6^, ^35^ HMPV viral load may be significantly correlated with disease course. In contrast, Jiang reported that coinfections were significantly more frequent among patients with a low than a high viral load. Previous studies^15^, ^26^ reported no difference in the clinical manifestations of children with single and multiple infections. In our study, patients infected with HBoV only were older (26.05 ± 31.22 months) than those infected with HBoV and other respiratory viruses (15.89 ± 18.94 months) (*P* = .005). Analyzing the correlation between viral load and coinfections is problematic because viral load can change rapidly over the disease course; this might explain the conflicting results among studies. Evaluating the clinical significance of one or more coinfecting viruses is difficult.^34^

Viral load is an important determinant of the severity of LRTIs caused by HBoV and HMPV.^16^, ^36^, ^37^ Our findings indicated that a high viral load was not associated with more severe disease, in agreement with a previous study.^6^ However, the effect of viral load on disease severity remains controversial. Of the HBoV‐ and HMPV‐positive patients, 83.7% (220/263) and 85.9% (171/199), respectively, were coinfections, and the coinfecting viruses may have contributed to disease severity. Moreover, because the NPAs were obtained during different disease stages, precise quantification could be difficult. This might also explain the conflicting results among reports.

This study has the following strengths: a considerable duration, large number of patients, and comparison of the most common viruses. Its limitation includes the lack of a control group without clinical evidence of illness. Further studies should compare a symptomatic group with a control group and a symptomatic period with an asymptomatic period.

In summary, we present a prospective study of LRTIs caused by HBoV and HMPV. A further comprehensive and in‐depth study of the role of HBoV and HMPV in LRTIs in China is warranted.

## COMPETING INTEREST

The authors have no competing interests to report.

## ETHICAL APPROVAL

The process obtained families’ informed consent, and the study protocol was approved by The First Affiliated Hospital of Hunan Normal University, Changsha, China.
